# The long-lasting impact of unemployment on life satisfaction: results of a longitudinal study over 20 years in East Germany

**DOI:** 10.1186/s12955-020-01608-5

**Published:** 2020-11-09

**Authors:** Ernst Peter Richter, Elmar Brähler, Yve Stöbel-Richter, Markus Zenger, Hendrik Berth

**Affiliations:** 1 Division of Psychological and Social Medicine and Developmental Neurosciences, Research Group Medical Psychology and Medical Sociology, Technische Universität Dresden, Fetscherstr. 74, 01307 Dresden, Germany; 2 Department of Psychosomatic Medicine and Psychotherapy, University Medical Center, Universität Mainz, Untere Zahlbacher Str. 8, 55131 Mainz, Germany; 3 Faculty of Managerial and Cultural Studies, University of Applied Sciences Zittau/Görlitz, Brückenstraße 1, 02826 Görlitz, Germany; 4 Department of Applied Human Studies, University of Applied Sciences Magdeburg-Stendal, Osterburger Str. 25, 39576 Stendal, Germany

**Keywords:** Unemployment, Life satisfaction, Subjective well-being, Cohort, Saxon longitudinal study

## Abstract

**Background:**

Economic disruption in East Germany at the time of reunification (1990) resulted in a noticeable increase in unemployment. The present study provides data from a German cohort for over 20 years. The aim was to examine how the frequency of experiencing unemployment affects life satisfaction and whether their relationship changes over time.

**Methods:**

In the Saxon Longitudinal Study, an age-homogeneous sample was surveyed annually from 1987 to 2016. Since 1996, 355 people (54% female) have been examined for issues related to unemployment. Life satisfaction was measured with both the Global Satisfaction with Life Scale and the Questions on Life Satisfaction^Modules^ questionnaire.

**Results:**

In 1996, the participants were 23 years old and 50% of the sample was affected by unemployment. At all 16 different measuring points, participants who were never unemployed indicated higher life satisfaction than those who were once unemployed. The repeatedly unemployed consistently reported the lowest values of life satisfaction. In each year, there were significant differences with small to medium effect sizes.

**Conclusion:**

Our results support the notion that the adverse effects of unemployment on life satisfaction increase with the time spent unemployed. In 2016, only 2% of the cohort were currently unemployed, but differences between people with and without unemployment experience still exist. This indicates that the negative effect of the unemployment experience will last for a very long time. To the best of our knowledge, this is the first study that demonstrates the effect so persistently at so many measurement points for over 20 years.

## Background

Unemployment is strongly associated with an increased risk of morbidity, mortality, mental health problems, and lower life satisfaction levels. The topic of unemployment has evoked a growing interest in 2020. The COVID-19 pandemic, which began in spring 2020, led to a collapse of the global economy and a massive increase in the unemployment rate [1]. A recent analysis of more than 1 million health insured individuals in Germany showed that the long-term unemployed had a more than 80 percent higher likelihood of hospitalization due to COVID-19 infection than the employed [2]. There is also evidence of a link between unemployment and suicides. A longitudinal analysis between 2000–2011 indicated that the relative risk of suicide associated with unemployment has increased by about 20–30% [3]. There was a massive increase in unemployment after the German reunification in 1990. The restructuring of the former East German economy led to the closure of many state-owned enterprises, resulting in massive job losses. The following unemployment rates were much higher than in West Germany. Despite considerable political efforts, these differences remain a social reality to this day. According to federal statistics, over 2 million people (5.3%) were unemployed in February 2017 in Germany. The differences between the East German states (7.0%) and the old West German states (4.9%) are significant [4].

Since 1990, almost all citizens of the East German states had experienced unemployment either themselves or within their family and acquaintances. The experience of unemployment in East Germany was a mass phenomenon with profound consequences [5]. A large number of studies have reported various links between unemployment and adverse psychological reactions, such as a higher number of mental disorders or more global aspects of daily life, such as impaired life satisfaction [6, 7]. Earlier studies concluded that the non-monetary effect of unemployment is much higher than the impact of the associated loss of income [8]. The consequences of unemployment include loss of social contact and identity, along with reduced self-esteem.

Life satisfaction can be defined as the cognitive aspect of subjective well-being and refers to the global assessment of the quality of life [9].

Hahn et al. [10] examined 908 individuals from 3 years before until 3 years after becoming unemployed. The results showed that experiencing unemployment leads to a significant decline in life satisfaction. Even when people found employment again, life satisfaction stagnated at a low level for many years after the period of being unemployed [11, 12].

While the effects of unemployment have been extensively investigated in cross-sectional studies, only a few longitudinal studies on the association of unemployment and life satisfaction have been conducted [13]. The present study provides data from a German cohort for over 20 years. We annually examined the link between life satisfaction and the frequency of experienced unemployment by using a large cohort from the Saxon longitudinal study. We want to investigate how the frequency of experienced unemployment affects life satisfaction and whether this relationship changes over time. Furthermore, we performed stepwise linear regression analyses in 1996 and 2016. We want to investigate whether life satisfaction is more strongly affected by current unemployment and its consequences or by the experience of past unemployment. Besides, we were interested if there were differences between the years 1996 and 2016 regarding the prediction of life satisfaction.

## Methods

### Sample

The Saxon Longitudinal Study (“Sächsische Längsschnittstudie”) [14–18] started in 1987 in the former German Democratic Republic (GDR). A sample (*N* = 1281) of 14-year-old students was selected as a representative group for the East German cohort of 1973. The sample was age-homogeneous because all the participants were in the eighth grade.

They were interviewed repeatedly until spring 1989. After the third survey in spring 1989, 587 of these participants agreed to continue participating in the study. The study continues after the German reunification until today. The Ethics Committee of the Technische Universität Dresden, Germany, approved the study protocol (No. EK8012011). The main topics of the study were political and social questions; for example, questions related to the long-term development of the GDR citizens’ socialization, their experience of the German reunification, and the changes in their living conditions. Since 1996, research into the consequences of unemployment has been a further focus of the study [19, 20].

In 2016, the 29th survey was conducted. On average, the 270 respondents in the 29th survey were 43 years old (53% female) and 77% had children. In 2016 the response rate was 46% based on the 587 people who had agreed to continue participating in 1989. Most of the respondents completed their vocational training; only 2% had no completed vocational training. Further information on the participants in the 12th (1996) and 29th (2016) surveys is provided in Table 1.

**Table 1:** Characteristics of the sample in 1996 and 2016

Characteristics	1996		2016	
	*n*	%	*n*	%
Total sample	355		270	
Mean age	23		43	
Female	192	54	144	53
Having children	46	13	209	77
Times of unemployment				
None	179	50	81	30
One time	115	33	82	30
Several times	61	17	107	40
Type of occupation				
Unemployed	38	11	6	2
Student	84	23	0	0
Home keeper	24	7	5	2
Blue-collar worker	77	22	48	18
White-collar worker	97	27	161	60
Self-employed	10	3	27	10
Public servant	0	0	16	6
Something else	25	7	7	2

### Questionnaires

Besides the socio-demographic parameters in the study, we have also collected a great deal of information on peoples’ experiences of the reunification and the transformation of East Germany [14–17]. Unemployment data were recorded by asking: “How many times have you been unemployed since 1990?”. Possible answers were “none”, “once”, and “several times”.

Furthermore, since 1996, the life satisfaction of the respondents has been measured annually with a self-developed, single-item scale—the Global Satisfaction with Life Scale (G-SLS). In the G-SLS, the respondents were asked to provide an answer to the question, “Taking all together, how do you assess your current life situation? With my life situation, I am.…” (in the original German: “Wie schätzen Sie—Alles in allem—Ihre gegenwärtige Lebenssituation ein? Mit meiner Lebenssituation bin ich…”). Participants answered on a symmetrical 5-point Likert scale ranging from 1 “very satisfied” to 5 “not satisfied at all”.

The FLZ^M^ (Questions on Life Satisfaction^Modules^, in the original German: Fragen zur Lebenszufriedenheit^Module^)) is a valid and widely used questionnaire to assess general life satisfaction. This questionnaire covers eight domains of daily life (e.g., family, work, health), and the respondents were asked to rate the subjective importance and their immediate satisfaction with each domain [21]. The Saxon longitudinal study has many different topics. For economic reasons, we therefore only used the single-item scale (G-SLS) to measure life satisfaction at all points in time. The longer, well-established questionnaire FLZ^M^ was only used at three points in time (2003, 2005, 2016). To check the validity of the G-SLS, we calculated the Pearson correlation with the well-established FLZ^M^. Table 2 shows the correlations of G-SLS with the other method of measuring life satisfaction. In our sample, the G-SLS correlates strongly with the well-established questionnaire FLZ^M^, which indicates a high convergent validity. The correlations are negative because the single-item scale (G-SLS) is inversely coded, with higher scores indicating lower life satisfaction. Whereas in the FLZ^M^, higher scores indicate higher life satisfaction.

**Table 2:** Pearson correlations of measurements of global life satisfaction

	G-SLS	
	*r*	*N*
FLZ^M^ (2003)	− 0.50***	409
FLZ^M^ (2005)	− 0.48***	371
FLZ^M^ (2016)	− 0.40***	268

***p < 0.001

### Statistical analysis

The data were analyzed using SPSS software version 25 [22]. The mean values and the standard deviations (*SD*) for life satisfaction measures (G-SLS, FLZ^M^) between groups (times of unemployment) were reported.

First, the data were analyzed using the Shapiro–Wilk test to determine whether they followed a normal distribution. The study found that the data did not follow a normal distribution. Therefore, the results were further analyzed nonparametrically using the Mann–Whitney-U-test and Kruskal–Wallis rank test. The Kruskal Wallis test is conservative and does not assume population normality, nor homogeneity of variance, and requires at least ordinal scaling of the dependent variable [22].

We calculated differences in respondents’ life satisfaction dependent on different frequencies of experienced unemployment (several times, once, none). Effect size eta squared (*η*^2^) was calculated for *H*-statistic to describe the magnitude of the effect [23]. Eta squared is defined as the proportion of variance in scores on the outcome variable that is predictable from group membership [24]. An effect size (ES) of *η*^2^ = 0.01 was defined as small, *η*^2^ = 0.06 as medium and *η*^2^ = 0.14 as large [25]. We set the significance level at *p* < 0.05 (two-tailed).

Two multiple linear regression analyses with stepwise inclusion were carried out. Depend variable was life satisfaction (G-SLS) in 1996 and 2016, regressed on gender, have children, type of occupation as dummy variables (unemployed, student, home keeper, blue-collar worker, white-collar worker, self-employed, public servant), and frequencies of experienced unemployment as dummy variables (none, once, several times). The stepwise inclusion method provided a measure of the relative effect of each predictor variable upon life satisfaction. These analyses started with the strongest predictor and added additional predictors if they explained significant additional variance in the dependent variable. The entered predictors were deleted in subsequent steps if they no longer contributed considerable unique predictive power to the regression. We set the inclusion criterion to *p* = 0.05 and the exclusion criterion to *p* = 0.10. The method terminated when no further variables were eligible for inclusion or exclusion. This minimized the possibility of entering two highly correlated predictor variables into the model. This method was used to identify the optimal set of predictors [26]. We reported standardized regression coefficients (*β*), which can be interpreted in the same way as regression coefficients.

## Results

### Gender aspects

In 1996, the participants were 23 years old and were surveyed on their unemployment experiences for the first time. Even then, 50% of the sample was affected by unemployment, 17% of the respondents several times and 33% once. Until 1990, the respondents were unemployed for a total of 6.5 months on average. In 1996, significant gender differences were found: The cumulative duration of unemployment was 4.9 months for men and 7.8 months for women since 1990 (*Z* = − 2.60; *p* = 0.01).

By 2016, 70% of the remaining sample was affected by unemployment, 40% several times, and 30% one time. On average, the cumulative duration of unemployment since 1990 was 10.9 months. In 2016 only descriptive differences in the duration of unemployment between men (9.0 months) and women (13.2 months) could be found, but those were no longer significant (*Z* = − 0.54; *p* = 0.59).

### The effect of unemployment periods and differences in life satisfaction

Table 3 summarizes the results in terms of life satisfaction (G-SLS) between the groups of never-, once- and several times unemployed from 1996 to 2015. At all 16 different points in time, people who were never unemployed reported higher life satisfaction than people who were once unemployed. Several times unemployed participants always reported the lowest values of life satisfaction.

**Table 3:** Life satisfaction (G-SLS) as a function of times of experienced unemployment between 1996 and 2015

	Times of unemployment						
	Total	Several	One time	None	*H*(2)	ES *η*^2^	*p* value
	Mean ± SD	Mean ± SD	Mean ± SD	Mean ± SD			
(N)		(n)	(n)	(n)			
G-SLS							
1996	2.3 ± 0.8	2.6 ± 0.9	2.4 ± 0.8	2.1 ± 0.7	22.87	0.059	< 0.001
(354)		(61)	(115)	(178)			
1998	2.2 ± 0.8	2.4 ± 0.8	2.3 ± 0.7	2.1 ± 0.7	15.75	0.038	< 0.001
(368)		(85)	(117)	(166)			
2000	2.1 ± 0.7	2.3 ± 0.8	2.2 ± 0.7	2.0 ± 0.6	18.40	0.042	< 0.001
(396)		(102)	(132)	(162)			
2002	2.2 ± 0.7	2.4 ± 0.8	2.1 ± 0.7	2.0 ± 0.6	21.38	0.042	< 0.001
(420)		(120)	(143)	(157)			
2004	2.3 ± 0.8	2.5 ± 0.8	2.2 ± 0.8	2.1 ± 0.7	28.26	0.064	< 0.001
(412)		(140)	(129)	(143)			
2006	2.3 ± 0.8	2.5 ± 0.7	2.3 ± 0.8	2.1 ± 0.7	15.29	0.035	< 0.001
(386)		(155)	(117)	(114)			
2007	2.2 ± 0.8	2.6 ± 0.8	2.1 ± 0.7	2.0 ± 0.7	42.07	0.113	< 0.001
(359)		(136)	(116)	(107)			
2009	2.2 ± 0.8	2.4 ± 0.8	2.1 ± 0.7	2.0 ± 0.7	27.37	0.070	< 0.001
(364)		(152)	(109)	(103)			
2010	2.3 ± 0.9	2.5 ± 0.9	2.2 ± 0.8	2.1 ± 0.8	10.86	0.028	0.004
(324)		(123)	(98)	(103)			
2011	2.3 ± 0.9	2.5 ± 1.0	2.2 ± 0.8	2.1 ± 0.8	15.22	0.036	< 0.001
(372)		(139)	(108)	(125)			
2012	2.3 ± 0.8	2.4 ± 0.8	2.2 ± 0.8	2.2 ± 0.7	11.77	0.028	0.003
(349)		(131)	(105)	(113)			
2013	2.3 ± 0.7	2.3 ± 0.7	2.3 ± 0.8	2.1 ± 0.6	6.58	0.014	0.037
(328)		(128)	(98)	(102)			
2015	2.2 ± 0.8	2.4 ± 0.8	2.2 ± 0.8	2.0 ± 0.7	17.47	0.047	< 0.001
(333)		(125)	(110)	(98)			

Higher scores in the G-SLS questionnaire indicate lower life satisfaction

In each year of data collection, there were significant differences between the three groups. The differences between the groups were highly significant almost every year (*p* < 0.001), all of small to medium effect sizes (*η*^2^ = 0.014–0.113).

Table 4 presents the results of the years 2003, 2005, and 2016, in which life satisfaction was measured with both the single-item scale G-SLS and the well-established questionnaire FLZ^M^. At all different points in time, there were differences in life satisfaction measured by G-SLS and also FLZ^M^ between the people who were never, once and several times unemployed (*p* < 0.02) with small to medium effect sizes [*η*^2^(G-SLS) = 0.055–0.099; *η*^2^(FLZ^M^) = 0.023–0.093]. The significance levels and effect sizes showed a high descriptive degree of convergence in their level between the two questionnaires.

**Table 4:** Life satisfaction in the years in which measured results from the G-SLS and FLZ^M^ questionnaires are available, depending on the times of experienced unemployment

	Times of unemployment						
	Total	Several	One time	None	*H*(2)	ES *η*^2^	*p* value
	Mean ± SD	Mean ± SD	Mean ± SD	Mean ± SD			
(N)		(n)	(n)	(n)			
G-SLS							
2003	2.2 ± 0.7	2.4 ± 0.7	2.2 ± 0.8	2.0 ± 0.7	24.59	0.055	< 0.001
(417)		(133)	(132)	(152)			
2005	2.3 ± 0.8	2.7 ± 0.9	2.2 ± 0.8	2.0 ± 0.6	39.72	0.099	< 0.001
(383)		(133)	(126)	(124)			
2016	2.2 ± 0.8	2.3 ± 0.7	2.2 ± 0.7	2.0 ± 0.8	13.49	0.043	< 0.001
(270)		(107)	(82)	(81)			
FLZ^M^							
2003	55.8 ± 29.4	47.4 ± 30.4	57.4 ± 29.7	62.4 ± 26.5	17.34	0.038	< 0.001
(409)		(130)	(131)	(148)			
2005	62.8 ± 30.7	49.5 ± 28.9	68.1 ± 29.2	71.2 ± 29.9	36.48	0.093	< 0.001
(375)		(128)	(125)	(122)			
2016	69.6 ± 35.1	62.6 ± 35.8	71.6 ± 33.5	76.8 ± 34.5	8.18	0.023	0.020
(268)		(105)	(82)	(81)			

Higher scores in the G-SLS questionnaire indicate lower life satisfaction, and higher scores in the FLZ^M^ questionnaire indicate higher life satisfaction

### Predictors of life satisfaction

Two stepwise linear multiple regression analyses were performed to identify which variables explained variance in life satisfaction in 1996 and 2016. The G-SLS score was used as a criterion variable of life satisfaction. Predictive variables were: gender, having children, different types of occupation, and the frequency of unemployment. The two multiple regression analyses showed that in 1996 and 2016, there was only one significant and consistent predictor of life satisfaction in each case. In 1996, currently unemployed as type of occupation (*β* = 0.81, *p* < 0.001) and in 2016, a repeated experience of unemployment (*β* = 0.24, *p* = 0.009) was associated with lower life satisfaction. The remaining predictor variables (gender, having children, and the different types of occupations) did not explain any additional variance in life satisfaction scores in our analysis.

## Conclusion

The results of the study show that people who have experienced unemployment in their occupational biography reported lower life satisfaction. This negative effect of unemployment is robust and persists for many years; it could be measured convergently with small to medium effect sizes by using two different questionnaires to assess life satisfaction. To the best of our knowledge, this is the first study that demonstrates the effect so persistently at so many measurement points for over 20 years.

Lucas et al. [11] and Winkelmann and Winkelmann [8] analyzed data from the German Socio-Economic Panel and found a similar pattern among people who have experienced unemployment, but only over a few measurement points. The negative effect was observed during unemployment and even during the period of re-employment. Our results support the assumption that the negative influence of unemployment increases with its frequency, as several times, the unemployed reported the lowest and the never unemployed the highest levels of life satisfaction. In order to avoid many multiple comparisons and alpha error inflation, we presented these differences only descriptively. The highly significant differences between people with and without the experience of unemployment could be shown consistently in all 16 surveys, both with the G-SLS and FLZ^M^ questionnaires for measuring life satisfaction. Our analyses showed that this effect has a small to medium effect sizes. This magnitude of the effect was also found in other analyses. A meta-analysis revealed that unemployment has a strong negative effect on the self-reported global life satisfaction with a mean medium effect size (*d* = − 0.44) [6]. Clark et al. [12] showed that life satisfaction is lower not only among people who reported a higher degree of previous unemployment but also among people who were currently unemployed (relative to the employed). We also found this association.

Our stepwise regression analysis showed that being currently unemployed was the only predictor for lower life satisfaction in 1996. It suggests that unemployment has a strong negative effect because gender, having children, frequency of experienced unemployment, or different types of occupation explained no further incremental variance. Other studies have shown that the other predictors from our regression may affect life satisfaction. An analysis of the World Value Survey of 34 countries revealed that people who had children showed significantly higher life satisfaction [27]. McKee-Ryan, Song, Wanberg, & Kinicki [6] reported in their meta-analysis that women are slightly less satisfied with their lives during unemployment than men. In Germany, unemployment rates have decreased significantly over the last 20 years. Since 1996, the unemployment rate in East Germany has almost been halved [28]. Unlike in 1996, in the regression analysis, being currently unemployed was not a significant predictor of life satisfaction in 2016. Only the repeatedly unemployed were significantly associated with life satisfaction in the stepwise regression. One possible explanation for the changed predictors could be that a five times smaller proportion of our sample was currently unemployed in 2016 than in 1996 (2% vs. 11%). In 2016, 98% of the cohort was employed, but differences between people with and without the experience of unemployment still existed. It indicates that the negative effect of unemployment experience will last for a very long time. Our results are constrained by some methodological limitations that should be considered in future studies. First, the Saxon longitudinal study is an investigation with more than 30 surveys by now. Due to non-compliance, not every person responded at all times, which leads to a different sample for different surveys. With the known limitation, we analyzed the data in the study only cross-sectionally. Due to a large number of different topics in the Saxon longitudinal study, we are only able to analyze the changes in life satisfaction by using a one-item scale in most surveys. It would be interesting to know whether similar results were found for other components of subjective well-being, for example, quality of life. Overall, our results support the idea that the adverse effects will cumulatively increase with the time spent unemployed and are persistent for many years. Therefore, it is crucial to see unemployment as a potential pathogenic factor. Future studies could investigate whether these effects still occur in older people, even if they are already receiving a retirement pension.

## Data Availability

The datasets generated and analyzed during the current study are available in the GESIS – Leibniz-Institut für Sozialwissenschaften in Mannheim (German) repository, https://www.bit.ly/sls-gesis and from the corresponding author on reasonable request.

## Abbreviations

ES: Effect size
GDR: German Democratic Republic
G-SLS: Global Satisfaction with Life Scale
FLZ^M^: Questions of Life Satisfaction
